# Intraoral Lipoma of the Cheek – A Case Report With a One-Year Follow-up and Review of Literature

**DOI:** 10.7759/cureus.10537

**Published:** 2020-09-18

**Authors:** Amal S Alharbi

**Affiliations:** 1 Department of Oral and Maxillofacial Surgery, Al Rass General Hospital, Alqassim, SAU

**Keywords:** oral tumors, adipocytes, benign, surgical excision

## Abstract

Lipomas are mesenchymal adipose tumors that are most common in the human body. However, they are rare in the oral cavity at an occurrence rate of 1% to 4% with male gender predilection. The case presented is of a 37-year-old male who presented with a large painless swelling on the right cheek region. The swelling was present for the past year and had aggravated in the previous one month, causing discomfort during mastication and speech. On clinical examination, a solitary non-fluctuating circumferential swelling on the right cheek was observed. A provisional diagnosis of lipoma was made based on the history and clinical examination, and it was decided to treat by surgical excision. A final diagnosis of lipoma was made based on histopathological analysis of the excised specimen. The patient at a one-week follow-up had recovered from his speech and chewing problems, and no recurrence was reported at a one-year follow-up.

## Introduction

Lipomas, also known as universal or ubiquitous tumor, are the most common benign mesenchymal neoplasms that can occur in any region of the human body [1]. They are composed of mature adipocytes, usually surrounded by a fibrous capsule [2], and are often seen in the subcutaneous and retroperitoneal spaces containing fat [3]. They commonly occur in the head and neck region; however, the occurrence in the oral cavity is rare with 1% to 4% incidence [4]. Lipomas occur especially in areas of fat accumulation, in particular, cheeks, followed by the tongue, the floor of the mouth, buccal sulcus and vestibule, lip, palate, and gingiva [4]. They typically occur in the fourth to fifth decades of life with peak incidence age at 40 years and with a male predilection. The etiology and pathogenesis of lipomas remain unclear, although mechanical trauma, endocrine disorders, obesity, hypercholesterolemia, radiation, and influences of chromosomal abnormalities are reported [5,6]. 

Typically, oral lipomas clinically present as slow-growing, deep-seated, soft painless masses with a typical yellowish color [7]. However, the location and size of the tumor may cause disturbances with speech and mastication [8]. Lipomas are usually treated with surgical excision because they have well-developed margins, and the rate of recurrence is 1% to 2% [9].

The aim of this article is to present a case of an adult male patient with intraoral lipoma that was treated by surgical excision with no recurrence or complications.

## Case presentation

**Figure 1:**
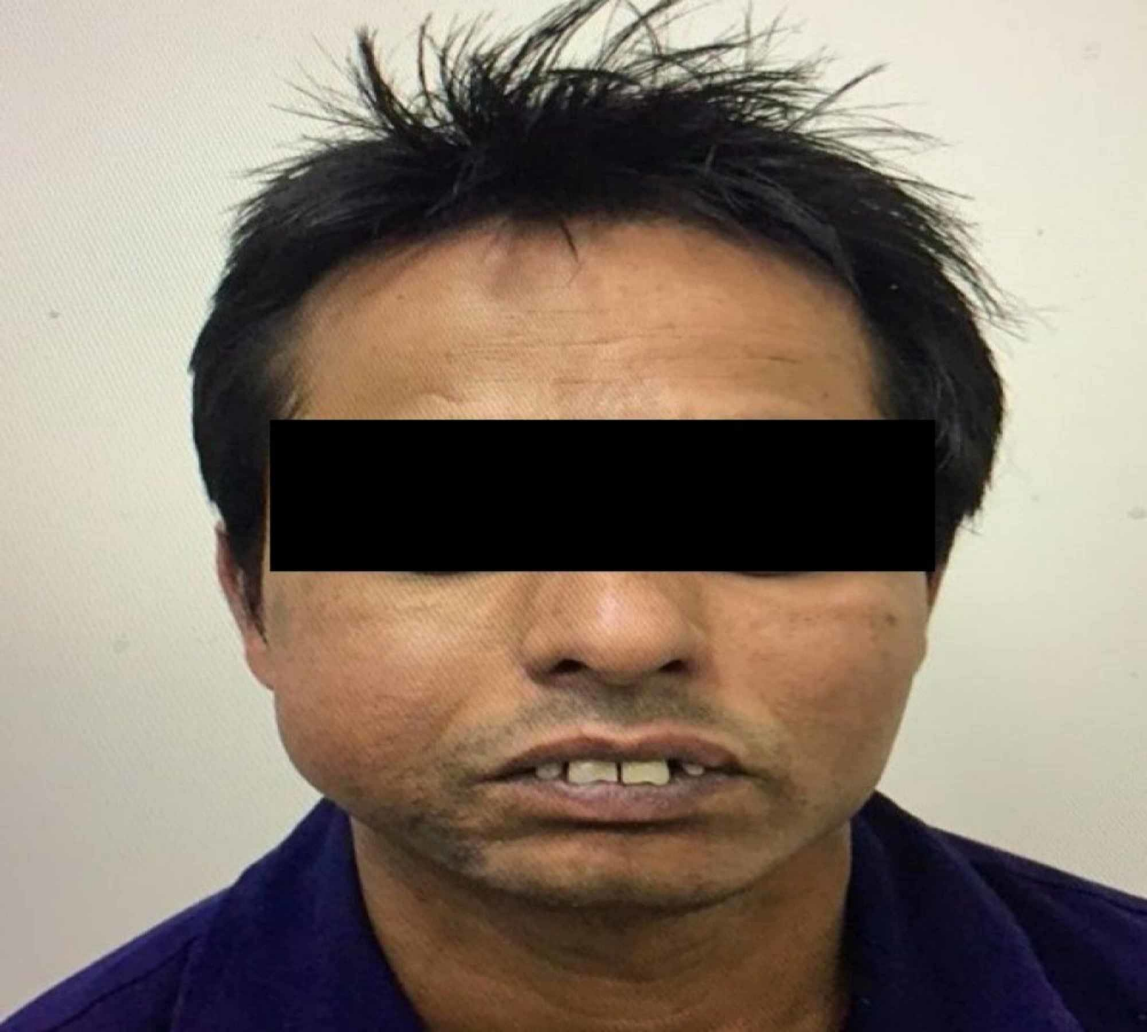
Extraoral photograph of the patient with the swelling

A 37-year-old male was referred from the emergency care to the Oral and Maxillofacial Surgery Department, Al Rass General Hospital, Al Qassim, Saudi Arabia. The patient presented with a large painless swelling on the right cheek region. He asserted that the swelling was present for the past year and had aggravated in the previous month causing serious discomfort during chewing and talking. The medical and family history was non-contributory to the chief complaint. The swelling was not associated with paresthesia, ulceration or discharge, recent fever within a month, and relevant loss of weight or appetite. The extraoral examination revealed a solitary non-fluctuating swelling in the right side of the mandible extending from the corner of the mouth to the angle of the mandible measuring 5 cm x 3.5 cm. Regional lymph was non-palpable. On intraoral examination, a solitary non-tender, non-fluctuating circumferential swelling attached to the right cheek located opposite to the first premolar, second premolar, and first molar was noticed (Figure 1). Intraorally, the swelling measured 5 cm x 4 cm. 

**Figure 2:**
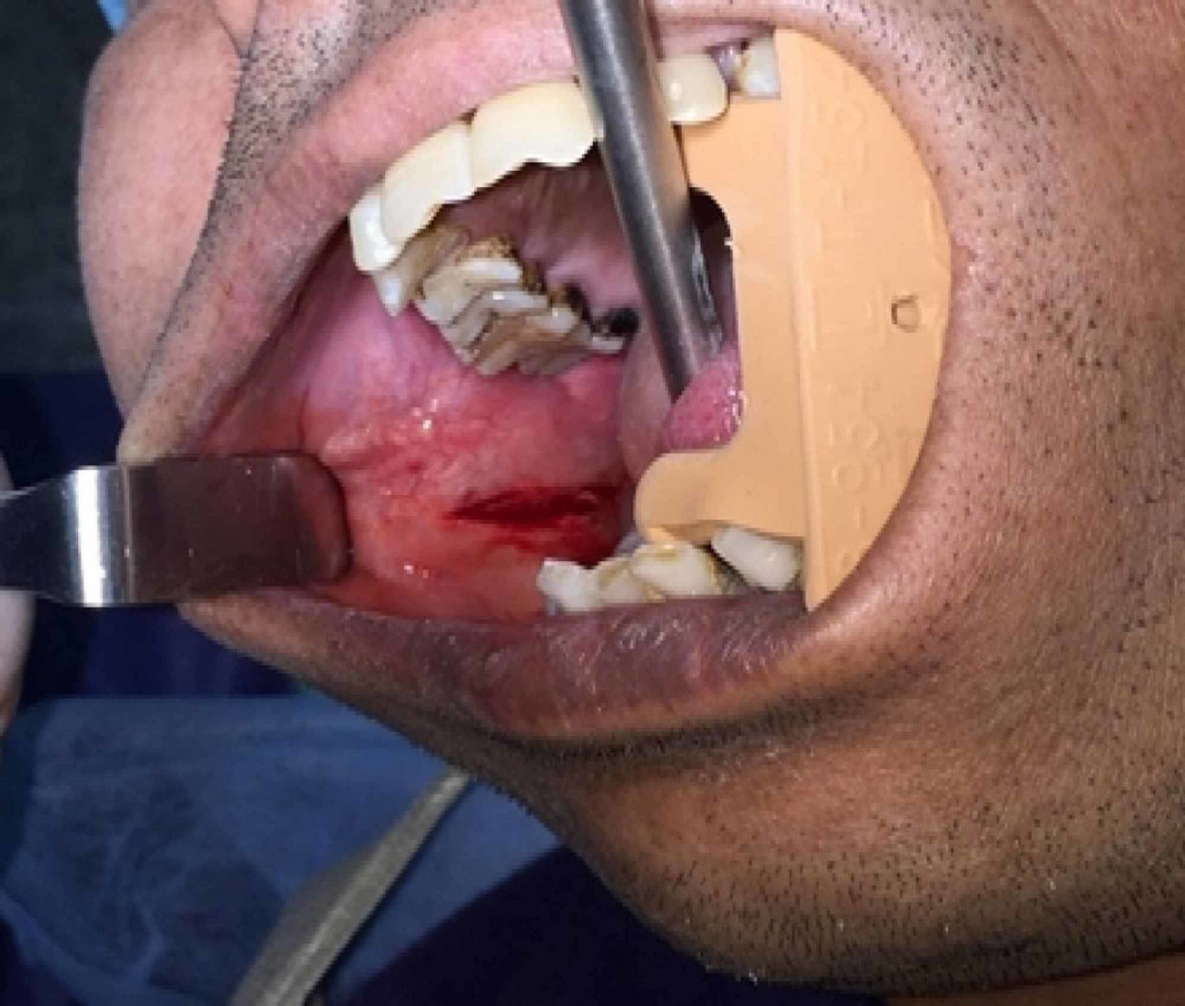
Intraoral photograph of the patient following surgical excision

Aspiration analysis did not offer any significant findings. A provisional diagnosis of lipoma was made based on history and clinical examination. The treatment plan included surgical excision under local anesthesia. The patient was explained about the surgical procedure with their advantages and risks for which the patient provided informed consent to undergo the treatment. A horizontal incision was performed in the occlusal plane region under infiltration anesthesia, and the mass was surgically excised with blunt dissection technique (Figure 2).

Gross examination

**Figure 3:**
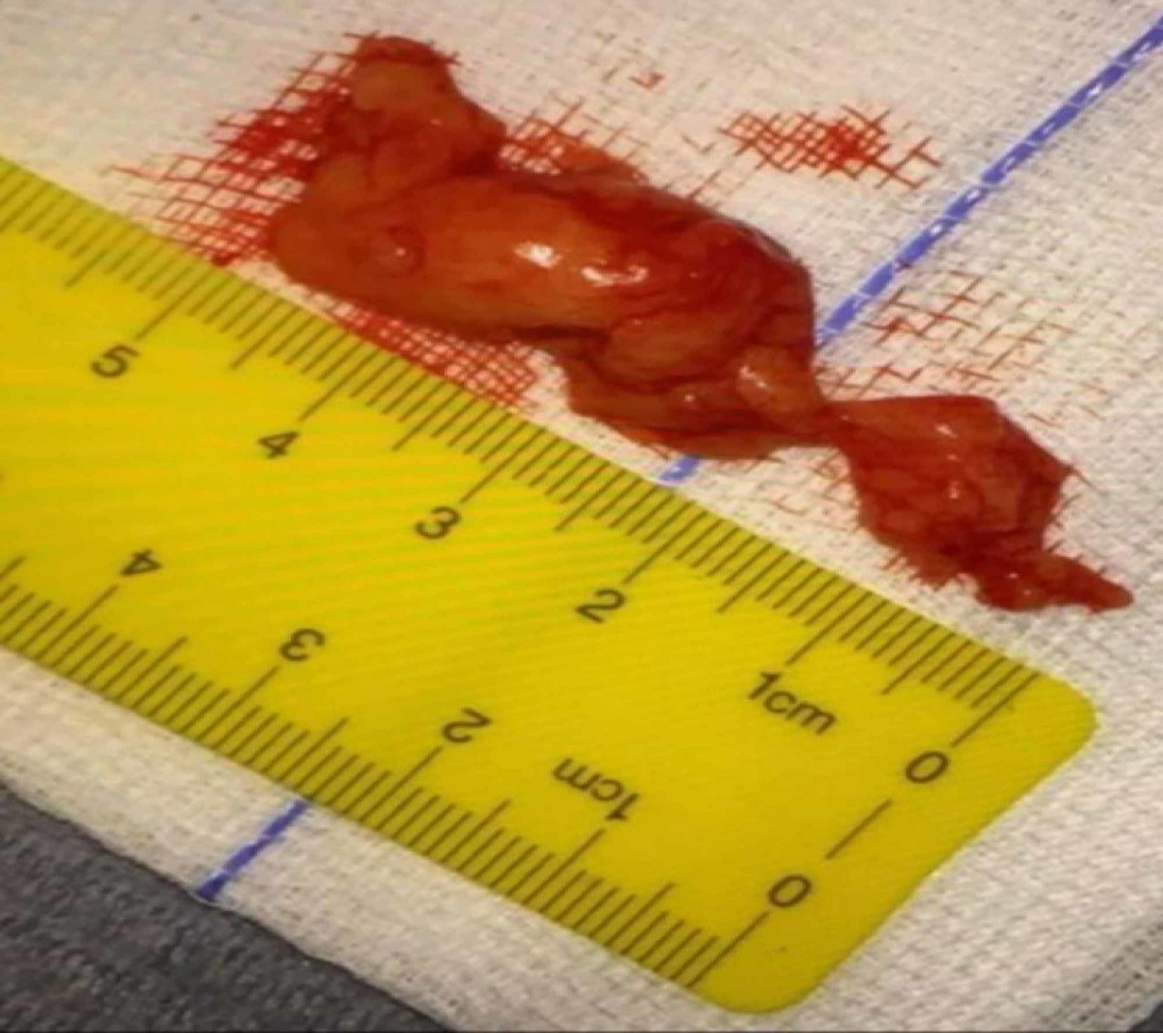
Surgically excised specimen

The excised specimen measured 5.4 cm x 4 cm in size (Figure 3) and was soft in consistency with a lobulated surface. The specimen was further sent for histopathological analysis. 

Histological examination

The histological analysis revealed lobules with vacuolated cytoplasm and peripheral flattened nuclei. The lobules were separated by thin fibrous bands showing delicate blood vessels. No metaplastic changes were observed, and the final diagnosis of lipoma was confirmed by the histopathologist.

**Figure 4:**
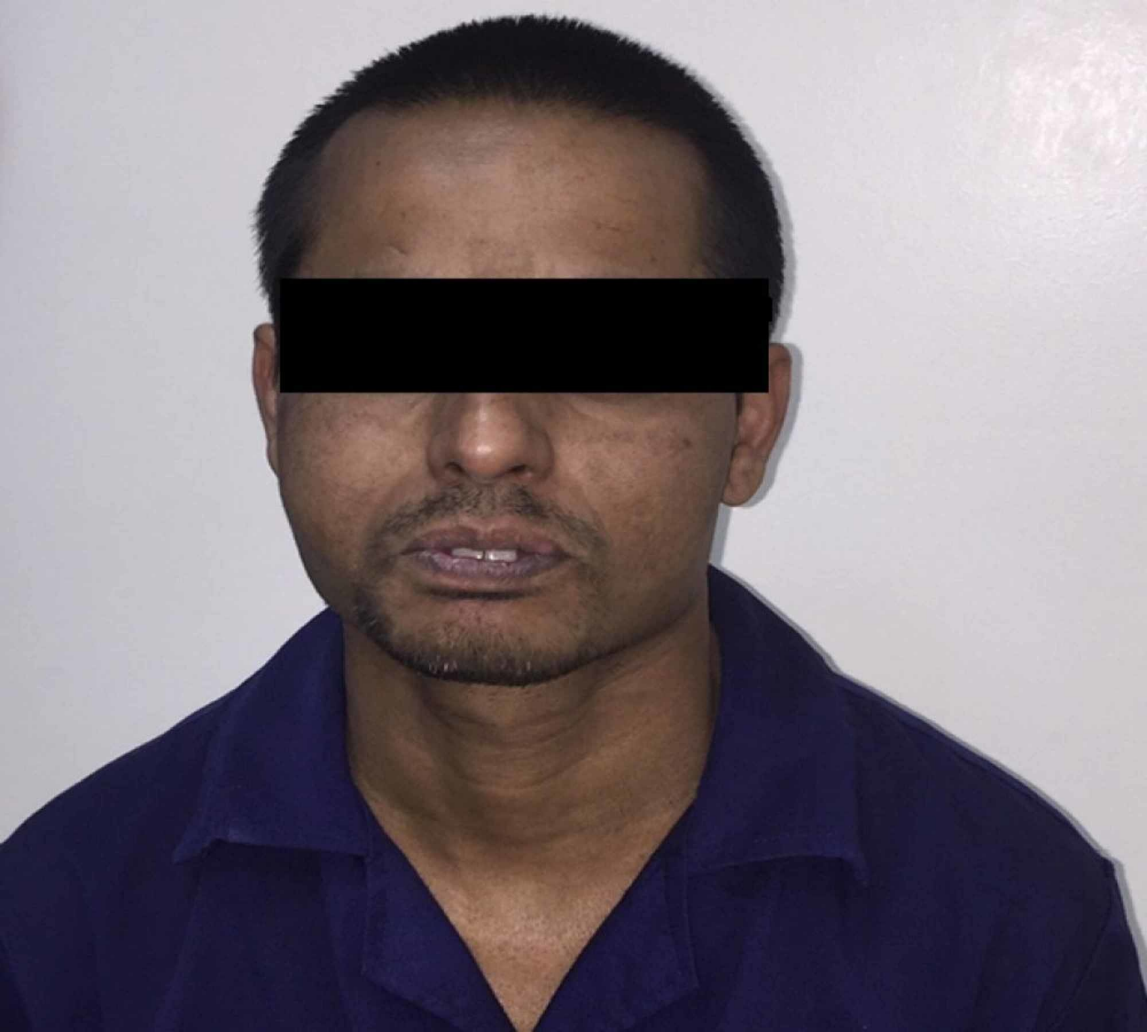
Extraoral photograph of the patient at one-year follow-up

The patient was prescribed analgesic (SOS) and discharged on the day of surgery, and was recalled after one week. The patient at a one-week follow-up had recovered from his speech and chewing problems. No complications such as paresthesia or recurrence were reported at a one-year follow-up (Figure 4).

## Discussion

Lipomas are a benign neoplasm of mature adipose tissue, which is commonly seen in the head and neck region [4]. Roux, in 1948, was the first person to describe oral lipoma in his review of alveolar masses, where he termed it as “yellow epulis” [10]. Intraorally, a lipoma is a rare entity with an occurrence rate of 1% to 4%. This evidence is further supported in a study by Furlong et al., in which the authors identified only 125 oral lipomas over the period of 20 years [11]. Few reports find equal gender distribution for the tumor [6,11], whereas some studies show a slight male predilection [12]. Although they are solitary, few syndromes such as Gardner’s syndrome, familial multiple lipomatosis, neurofibromatosis, Dercum’s disease, Cowden’s syndrome, and Proteus syndrome are associated with multiple lipomas [6,10,12].

Clinically, intraoral lipomas are usually slow-growing and asymptomatic. However, few reports associate intraoral lipomas with dysphagia and dyspnea apart from speech and mastication problems depending on the size and anatomical location [13,14]. In the present case, the patient had noticed the swelling a year before but had not considered treatment. However, he developed problems with normal functioning such as talking and chewing in the previous month, which made him seek treatment. The time frame when the patient noticed lipoma to seek medical attention ranges from 1 month to 10 years, with a mean of 2 years [9]. The excised specimen in this study was around 5.5 cm x 4 cm, which is fairly large; probably due to its growth over a year.

The exact etiology and pathogenesis of lipomas still remain unclear, but they are often associated with mechanical trauma, endocrine problems, obesity, hypercholesterolemia, radiation, chromosomal abnormalities, and diabetes [5,6,15,16]. However, in the present case, the patient did not have any history of mechanical irritation or trauma from sharp teeth. This is very important from both the clinician and the patient's view to prevent the recurrence of the lesion. 

Histologically, lipomas consist of mature adipocytes grouped in bundles displaying clear cytoplasm and eccentric nucleus separated by connective tissue septa [2]. They are morphologically identical to normal fat and can be differentiated by the presence of an enclosing thin fibrous capsule. Due to the similarities in the histological features between lipoma and normal fat, it is very important to consider tumors such as epidermoid cysts, pleomorphic adenomas, fibromas, thyroglossal duct cysts, ectopic thyrohyoid tissue, mucoepidermoid carcinoma, and oral dermoid and lymphoepithelial cysts in the differential diagnosis [7,10]. 

Although rare, the malignant transformation of lipoma has also been reported [17]. These malignant tumors are defined by areas of lipoblastic proliferation, cellular pleomorphism, myxoid differentiation, increased vascularity, and mitosis [7]. The treatment of choice for lipoma is surgical excision; however, the removal can be difficult in situations if they are positioned deeply. Recurrence or complications following surgical removal are rare [9] as also observed in the present case at a one-year follow-up.

## Conclusions

Lipomas are common benign tumors in the head and neck region, but their intraoral occurrence is uncommon. They typically grow slowly and are asymptomatic, and are the main reason why patients do not report and seek treatment. Nevertheless, the location and size of the lipoma at an indefinite time may cause discomfort during speech and mastication. Although non-surgical treatment is under consideration, surgical excision still remains the treatment of choice for this lesion.
